# COVID-19 stressor reduces risk taking: the role of trait interoception

**DOI:** 10.1007/s10339-023-01134-4

**Published:** 2023-03-28

**Authors:** Miguel Omar Belhouk-Herrero, Francisco Molins, Miguel Ángel Serrano

**Affiliations:** Department of Psychobiology, Universitat de València, Valencia, Spain

**Keywords:** Decision-making, Framing effect, Stress, Interoception, Covid-19, Alexithymia

## Abstract

The Framing Effect (FE) demonstrated that the way two alternatives are displayed affects people’s inclination to make a specific choice, showing a risk aversion when alternatives are displayed on positive frames and risk seeking in negative frames. Risk seeking in negative frames is closely linked to loss aversion. Moreover, classical research and the salience-of-losses hypothesis argues that stress may enhance the FE and loss aversion. Recent studies also suggest that the trait interoception and alexithymia could interact and moderate the framing susceptibility. However, experimental paradigms on stress could ignore variables such as threat perception. In this sense, COVID-19 pandemic has become a powerful real-life stressor in many countries. We aimed to study how real-life stressors influence decision-making under risk. A total of 97 participants were divided into a control (*n* = 48) and an experimental group (*n* = 49). The experimental group were exposed to a stressor manipulation, a 5 min COVID-19 lockdown documentary. Our results show that COVID-19-related stressors significantly decreased bet acceptance regardless of the frame, also reducing loss aversion. Moreover, interoception was a significant predictor of loss aversion under stress conditions. Our results do not support classical research on stress and FE.

**Supplementary Information:**

The online version contains supplementary material available at 10.1007/s10339-023-01134-4.

## Introduction

Daily life situations require decision-making processes. Despite the influence of the rational choice theory (Becker 1968; Bernoulli 1954; Deming, von Neuman and Morgenstern, 1945)—which argues that individuals select the best behaviour in order to maximize their utility regardless of the context—the Prospect Theory proposed by Kahneman and Tversky (1979) demonstrated that changes in the context could lead to a disruption in the risky choice consistency. This disruption can be induced by the Framing Effect (FE), a widely studied cognitive bias that suggest that the way different alternatives are displayed affects people’s inclination to make a specific choice. For example, several studies have shown a reversed risk preference when presenting the same option in different frames (positive vs. negative frames). In addition, it has been reported a risk seeking when the choice involves losses and a risk aversion in choices involving gains (Detweiler et al. 1999; Kühberger et al. 1999; Pinon & Gambara 2005; Tversky & Kahneman 1981). This frame-dependent inconsistency has been observed in hypothetical scenarios involving human lives (the “Asian Disease Problem”; see Tversky & Kahneman 1981) and also in economical risky choice tasks (de Martino et al. 2006).

Together with the FE, stress seems to be an important modulator of decision-making strategies. In fact, many real-life decisions are made under stress, and the decision itself could be a source of pressure. Previous research has shown a link between stress and a performance decrease in decision-making tasks (Starcke & Brand 2012; Wemm & Wulfert 2017). Furthermore, classical research found that higher arousal level leads to magnify the risk seeking on loss-domain choices and risk aversion on gain-domain choices (Porcelli & Delgado 2009; Ring 2015; Yamakawa et al. 2016). In other words, higher arousal increases FE. This could be due to the release of glucocorticoids and noradrenaline, which reduce intracellular signalling along prefrontal-amygdala circuits (Arnsten 2009; Yamakawa et al. 2016). In fact, under psychological stress conditions the amygdala activates hypothalamic and brainstem structures leading to an enhanced noradrenaline and dopamine inputs and impairing the prefrontal function. Since prefrontal cortex structures are involved in higher order decision-making and inhibition of impulsive behaviours, acute stress could lead individuals to perform a biased and non-reflexive strategies, especially in loss frames (Arnsten 2009). This line of evidence is in accordance with the salience-of-losses hypothesis (Metz et al. 2020), which argues that the activation of the salience network in stress conditions should increase the psychological impact of losses. Nonetheless, it is important to emphasise that the effects of acute stress on decision-making can be moderated by other emotional and psychophysiological variables, such as the trait interoception and alexithymia. Interoception—the ability to process and integrate inner body signals (Chen et al. 2021)—has recently become a new topic of investigation even though the Somatic Marker Hypothesis (Damasio 1996) was formulated few decades ago. Some evidence shows that interoceptive awareness could predict decision-making performance under risk (Salvato et al. 2019). In the case of alexithymia, it seems to reduce the FE by blocking the incorporation of emotional feedback into decision-making (Shah et al. 2016a, b). It is known that alexithymia and interoception have opposite directions in both emotional awareness and FE susceptibility (Laloyaux et al. 2015; Shah et al. 2016a, b; Sütterling et al. 2013). Moreover, a recent study by Manzoor et al. (2021) showed that interoception and alexithymia could interact and predict low rates of FE sensitivity.

It is important to notice that most of the research makes use of artificial elements or paradigms to elicit emotional responses rather than real-life-related ones. This can lead to a bias ignoring cognitive variables such as threat perception (Lazarus 1966; Lazarus & Cohen 1977; Lazarus & Folkman 1984). For example, Henze et al. (2017) found that the Trier Social Stress Test (TSST)-induced similar neuroendocrine responses to that of a real-life oral exam, but instead the naturalistic stimuli had a more pronounced psychological impact in the sample. In this context, COVID-19 pandemic has become a powerful stressor since its inception (Lee et al. 2020). Concretely, in Spain, almost 20% of the population suffered high levels of anxiety and severe depressive symptoms during lockdown, being young adults the most affected demographic cluster (Becerra-García 2020; Universidad Complutense de Madrid, 2020). In addition, governments, citizens and health officials had to make decisions where the lives of others were at stake, making the pandemic an opportunity to study the effects of real stressors on risky choice tendency.

Based on all the above, the present study aims to investigate how negative COVID-19-related emotions can modulate decision-making processes and how emotional and psychophysiological variables such as interoception and alexithymia can moderate this relationship. Using the FE paradigm and following the salience-of-losses paradigm, it is expected that the exposure to a COVID-19 emotional stressor would increase FE in a risky choice task. According to previous evidence, we also expected an interaction between interoception and alexithymia mediating the influence of perceived stress in FE scoring.

## Method and materials

### Participants

A total of 97 participants (women: *N* = 85; age: *M* = 21.74, *SD* = 2.82) were randomly assigned to a control (*n* = 48) or experimental group (*n* = 49). Importantly, our sample mainly consisted in students who were recruited from psychology’s faculty in the University of Valencia. Previous experiments (Shah et al. 2016a, b; Starcke et al. 2008) report a large effect sizes between stress and decision-making processes (*d* = 0.96) and between interoception and FE (*d* = 1.77)*.* Given our sample size, a sensitivity test revealed a sensitivity of up to *d* = 0.269 to perform a multiple linear regression model with interoception and alexithymia as predictors (power = 90%, *α* = 0.05; sensitivity was calculated using *pwr* package on RStudio). Prior to the experiment, participants gave their informed consent. All procedure was conducted in accordance with the Declaration of Helsinki.

### Measures

#### Framing effect task

An economical framing task adapted from De Martino et al. (2006) was used to evaluate participants’ decision-making under risk. The task consisted of a list of 48 items in which the participants were asked to choose between two economical options. Firstly, a message appeared indicating the initial amount of money (€20, €25, €40, €50, €75, €80 and €100). Next, they had to choose between a “sure” or a “gamble” (risky) option. The “sure” options were set out in a positive (“You keep €50”) and in a negative frame (“You lose €30”). The “gamble” option proposed a probability of winning or losing certain amount of money (20%, 40%, 60% and 80%). For example: “A. You keep/lose €30; B. Gamble, knowing that there is a 60% chance of keeping everything and a 40% chance of losing everything”.

Both frame conditions and probability of win/lose were fully balanced in every starting amount condition. In order to avoid possible bias (Barkan & Busebeyer, 2003; Tversky & Kahneman 1992; Xue et al. 2011) there was no feedback given referring to economic balance after each choice.

Two possible ways to analyse the FE task are available: obtaining the percentage of bet acceptance in each frame and calculating the difference between gambling in loss and gain frames (that is, the size of Framing Effect).

#### Interoception

The *Multidimensional Assessment of Interoceptive Awareness V2* (MAIA-2; Mehling et al. 2018) was used to evaluate interoception. MAIA-2 includes 37 items on eight subscales in which participants must indicate the level of agreement with each statement in a scale ranging from 0 (never) to 5 (always). Cronbach’s Alpha for MAIA-2 in our sample was *α* = 0.92.

#### Alexithymia

To evaluate alexithymia, we applied the 20-item-based *Toronto Alexithymia Scale* (TAS-20; Bagby et al. 1994) in its Spanish version (Martínez-Sánchez 1996). TAS-20 is a self-report questionnaire answered in a 5-point Likert scale (1 meaning “completely disagree” and 5 meaning “completely agree”). Cronbach’s Alpha for TAS-20 in our sample was 0.78.

#### COVID-19 pandemic psychological impact

In order to explore the psychological effects of COVID-19 pandemic, we used two scales from the *Coronavirus Psychological Impact Questionnaire* (CIPC; Sandín et al. 2020). Particularly, we selected the *Experience with The Coronavirus* (ECOVI) and the *Distress Scale* (ED; *α* = 0.93, *ω* = 0.93). The aim of the ECOVI was to obtain data regarding the infection level of the participants and their close relationships (infections, hospitalizations and deaths), whereas ED evaluates negative emotional experiences and sleep problems during the Spanish lockdown.

#### Emotional variables

To evaluate emotional variables, we assessed the General Health Questionnaire (GHQ-12; Goldberg & Williams 1988) to explore psychological morbidity in our sample. A 3-item Self-Assessment Manikin questionnaire was also used, asking for perceived levels of happiness, arousal and dominance (Lang et al. 1993). In addition, we designed an *ad hoc* 1 to 10 Likert scale question for this study asking about subjective stress. This question asked: “What is your current stress level?”

### Procedure

Due to COVID-19 restrictions and the cumulative incidence in the moment of the methodological designing—exceeding 1000 per 100.000 cases in 14 days—the experiment was conducted in an online modality. It was divided in two phases. The first phase consisted in two different online questionnaires. The first questionnaire aimed to obtain sociodemographic information about participants, a mental health indicator measured with the *General Health Questionnaire* (GHQ-12), interoception and alexithymia punctuations (MAIA-2 & TAS-20) and COVID-19-related information (ED & ECOVI scales). The second questionnaire included the framing task. In order to standardize the procedure as much as possible, a PDF file was sent to the participants with step-by-step instructions on how to proceed. Lighting advices and links to assess the questionnaires were also included.

In the second phase, both control and experimental groups indicated their emotional state with a Self-Assessment Mannequin (SAM)-like questionnaire and their perceived stress levels. Next, they had to watch a video file. The experimental group watched a 4:17 min piece containing shocking images regarding the Spanish lockdown. Silent and empty streets images, collapsed intensive care units and severely ill patients are a few examples (full video file is available at Supplementary Materials). The control group watched a documentary clip (4:56 min) on how to produce marbles. Once they had finished watching the video, participants reported their emotional state and they had to perform the post-test framing task. As in the first phase, participants received a PDF file with step-by-step instructions, links and detailed directions on lighting, comfort, recommended distance from the computer and audio details to adjust the viewing conditions of the audio-visual content. To allow participants to meet the requirements, they were given a time window in which to carry out each phase the experiment.

Importantly, all the procedure including both phases were conducted during March 2021, just one year after the third wave of the pandemic in Spain which was the second most lethal after the first and at the start of vaccination in Spain for older people.

### Statistical analyses

Kolmogorov–Smirnov test was conducted to check normal distributions in our variables. To ensure that the video file evoked emotional responses, data regarding perceived stress, happiness, arousal and dominance was analysed performing a repeated measures ANOVA with group as a between-participants factor. To explore decision-making in the FE task, a repeated measures ANOVA was used to compare bet acceptance in each frame. Finally, interoception, alexithymia and its relationship with stress and FE were analysed performing a multiple linear regression model. Regression analysis were performed by each group and frame condition.

All analyses were performed using JASP v0.14.1.0 with α significance level set at 0.05.

## Results

Table 1 shows descriptive statistics. Participants who exceeded ± 3 standard deviations were filtered out as outliers. Kolmogorov–Smirnov normality test was performed and variables that did not follow a normal distribution were converted using a log (y + 1) transform.

**Table 1:** Descriptive statistics (*N* = 86)

	Control		Exp	
	M	SD	M	SD
Interoception	2.95	0.64	2.74	0.71
Alexithymia	43.33	8.85	44.98	10.29
Perceived stress pre	6.81	1.67	6.21	1.88
Perceived stress post	5.76	1.61	7.09	1.72
Bet acceptance gain frame pre (%)	36.90	14.62	38.92	17.05
Bet acceptance loss frame pre (%)	43.55	17.05	47.73	18.03
Bet acceptance gain frame post (%)	44.66	11.97	31.06	17.80
Bet acceptance loss frame post (%)	52.46	15.79	38.54	20.70
FE Pre	6.65	8.78	8.81	11.19
FE Post	7.80	9.27	7.48	12.04

*M* Mean; *SD* Standard deviation

Control and experimental groups were equally distributed in age, gender and socio-economical variables. No differences were also found in their experience regarding COVID-19 pandemic.

Going further on their COVID-19 experience, most of the participants reported not having received a diagnosis nor experiencing compatible symptoms (87.7%), while none of those who received a diagnosis or experienced symptoms needed to be hospitalised. 61.3% of our sample reported having a close friend or familiar with COVID-19 diagnosis of which 32.3% needed health care assistance. Finally, 9.4% of our sample informed having a close friend or familiar who died because of the infection.

### Effects of the stressor on psychological variables

**Fig. 1:**
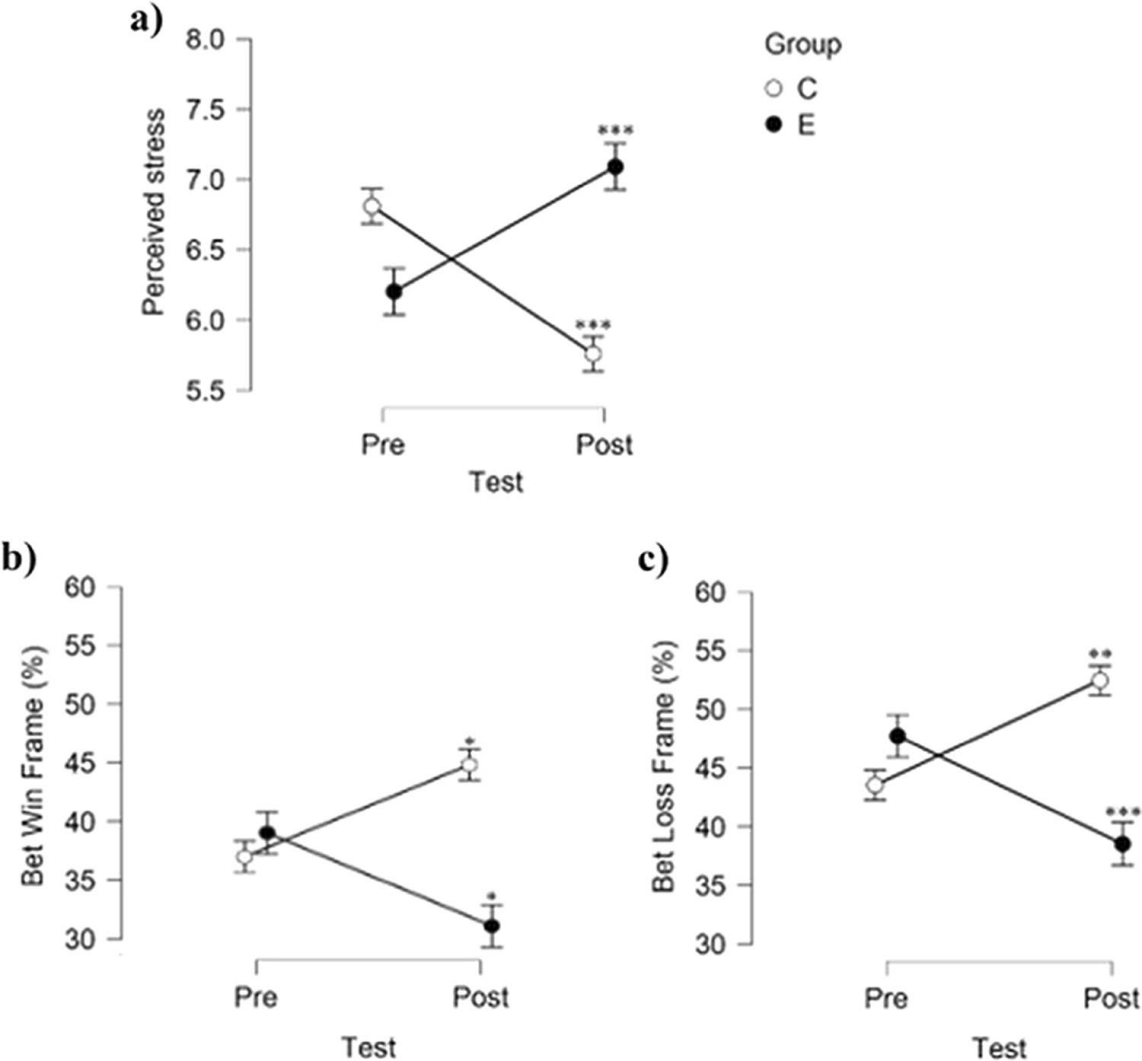
Effects of video file on perceived stress and bet acceptance. *Note* Figure shows the effect of the video file on perceived stress and bet acceptance in the FE task. **a** Experimental group (E, black) significantly increased their perceived stress rate, whereas control group (C, white) decreased. **b** Bet acceptance in the FE task on gain domain. **c** Bet acceptance in the FE task on loss domain. * *p* < 0.05; ** *p* < 0.01; *** *p* < 0.001

Group effects were found between pre and post-test in perceived stress (F(1, 84) = 44.318, *p* < 0.001, *d* = 0.565), happiness (F(1,84) = 77.197, *p* < 0.001, *d* = 0.824) and dominance (F(1, 84) = 15.597, *p* < 0.001, *d* = 0.523) performing a two-way repeated measures ANOVA with Bonferroni correction. Perceived stress increased in the experimental group after watching the video file (*p* < 0.001) whereas it decreased in the control group (*p* < 0.001) (see Fig. 1a). On the other hand, happiness and dominance levels significantly decreased in the experimental group (*p* < 0.001).

### Framing effect

No between group differences were found at pretest in any frame. In general, participants tended to take more risks in the loss frames than in the win frames (F(1, 84) = 50.786, *p* < 0.001, *d* = 1.546), confirming that FE was present in our sample. Repeated measures ANOVA showed a group effect within subjects between tests in both frames (F(1, 84) = 21.682, *p* < 0.001, *d* = 0.500 and F(1, 84) = 32.470, *p* < 0.001, *d* = 0.505 for gain and loss frame, respectively). More specifically, Bonferroni correction indicated that the experimental group increased their risk aversion after being exposed to the stressor since their bet acceptance decreased in win frames (*p* < 0.05) as well as in the loss frames (*p* < 0.001). Conversely, control group increased their bet acceptance in both win (*p* < 0.05) and loss frames (*p* < 0.01) (see Fig. 1b and c).

### Interoception and alexithymia

**Fig. 2:**
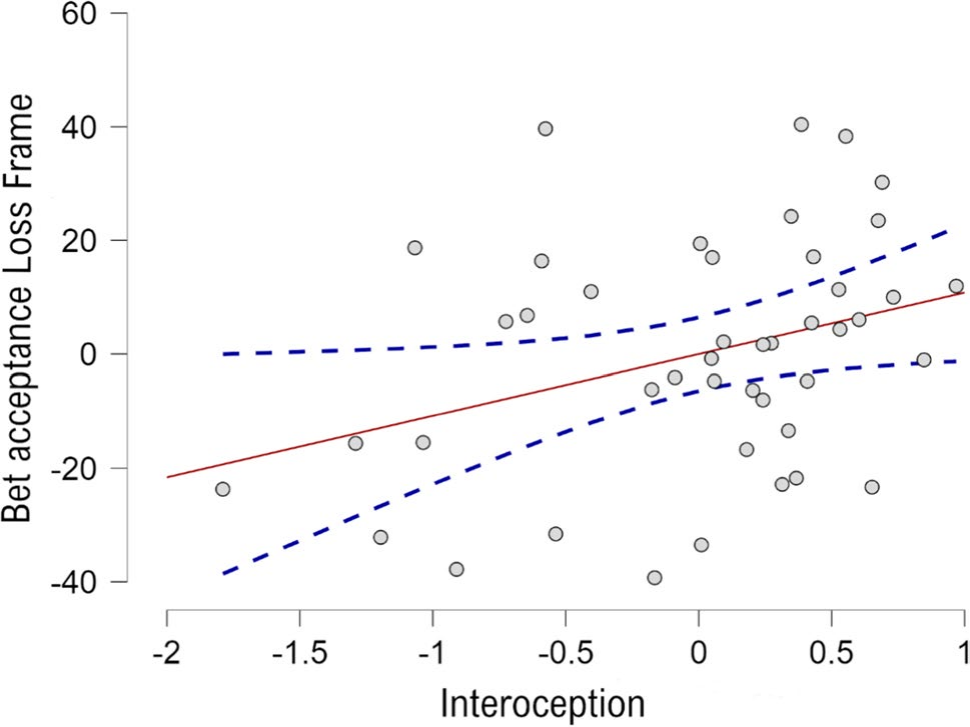
Relationship between interoception score and bet acceptance on loss frame under stress. *Note* Figure graphically represents the relationship between interoception and bet acceptance since interoception become a significant predictor in the experimental group after watching the video file. For each unit of interoception scoring, the bet acceptance increases in 13,37%

Four multiple linear regressions were performed to explore how interoception and alexithymia predict bet acceptance in each frame and condition. In the case of control group neither alexithymia nor interoception explained significantly decision-making under both frames (Gain Frame: F(2, 47) = 0.102, *p* = 0.904; R^2^ = 0.004; Loss Frame: F(2, 47) = 0.858, *p* = 0.431; R^2^ = 0.037). In the case of experimental group, a significant regression equation was found at loss frame (F(2, 46) = 4.803, *p* = 0.013; R^2^ = 0.173) (see Fig. 2). Nonetheless, only interoception was a significant predictor of bet acceptance (*B* = 0.430, *p* < 0.01). Bet acceptance in loss frames under stress conditions were 13.37% higher for each interoception mean unit increase. Alexithymia was not significant (*B* = 0.069, *p* = 0.83). In the case of gain frame, neither interoception nor alexithymia emerged as significant predictors (F(2, 48) = 2.001, *p* = 0.147; R^2^ = 0.080).

## Discussion

The results obtained in the present research shown that a COVID-19-related emotional stressor can modulate decision-making on risky choices. Classical theories about the effect of stress on FE argues that higher levels of stress could increase risk aversion in gain frames and risk tendency in loss frames. Our data do not support this. Instead, our results reject the salience-of-losses hypothesis and suggest a decreased risk seeking in both frames after being exposed to a stressor making individuals more conservative in any type of risky choices. In other words, stress and higher arousal may reduce loss aversion.

Two different neural systems are reported to be related with risk tendency and risk aversion—closely linked to loss aversion (Kahneman et al. 1991). The first one, the *appetitive system*, involves striatal structures and frontal regions such as the ventromedial, ventrolateral or dorsolateral prefrontal cortex (Sokol-Hessner et al. 2013; Tom et al. 2007). These structures are associated with reward seeking and could explain how individuals tend to make more conservative choices on gain frames. The second neural circuit, the *aversive system*, involves structures such as the amygdala or the posterior insular cortex (LeDoux 2012). In this sense, the aversive system has become a key feature providing the neural basis of loss aversion and explaining how classical research on FE found an increased risk tendency in loss frames under stress conditions. However, the influence of both systems does not seem to be as simple. It is known that the amygdala can also be activated by potential gains and that higher rewards or incomes can be processed as a potential loses (Molins & Serrano 2019). Moreover, we cannot define the role of these neural circuits only in terms of “appetitive” or “aversive” stimuli. Decision-making results from computing processes where a particular alternative is valuated regarding its cost–benefit subjective balance (Croxson et al. 2009). Thus, it should not be contradictory that different sources of stress affect decision-making differently. In fact, our results are in-line with recent previous evidence and provides further evidence on how emotional stress reduces risk seeking and loss aversion, being in accordance with a new body of evidence (Molins et al. 2021; Margittai et al. 2018). It is also important to notice that we used a real-life-related source of stress in our experiment. That is, our audio-visual stimulus is not a real-life stressor per se but it can potentially prime participant’s personal experiences regarding the pandemic. In this sense, in a COVID-19-related context a particular amount of loss may be processed as a potential gain compared to the risk of lose a higher amount of resources. Furthermore, the reduction in loss aversion supports the reward-alignment hypothesis (Metz et al. 2020), whereby the reduction in loss aversion could be induced by an activation of the reward system. The reward-alignment hypothesis has an empirical evidence since Mather and Lightball (2012) found an enhanced dopamine activity under stress conditions in reward-related central structures. Decision-making, ultimately, would be determined by the balance of the activation of these different neural circuits and the posterior cost–benefit computation in the prefrontal cortex. Both the nature of the stressor and the alignment hypothesis provide a coherent explanation for the results obtained in our study.

In addition, the observed relationship between interoception and decision-making is in accordance with the above. Linear regression showed that interoception is a significant predictor of bet acceptance in loss frames after being exposed to the stressor. The more interoception rate, the more bet acceptance in loss frames. Insular and cingulate cortices—also involved in the *aversive system*—are a central hubs for processing and integrating inner body signals (Chen et al. 2021). Consequently, we hypothesise that higher interoceptive awareness could break the loss aversion reduction in balancing the individual cost–benefit valuation towards the *aversive system*. Based on previous evidence (Manzoor et al. 2021), we expected higher rates of alexithymia to be related to less framing susceptibility. We have not found this effect, but subsequent studies should inquire in this concern.

Nevertheless, we cannot forget to mention a few limitations of our study. Firstly, our sample was small, considering test performed, and mainly composed by women (*N* = 85). There is evidence that risk taking may differ between men and women (Mather & Lightball 2012) and between different hormonal responses during acute stress (Alacreu-Crespo et al. 2019). In this regard, it might be interesting to replicate our results with a sex-balanced sample and controlling endocrine responses. Secondly, our control group did not behave as a real control group. Although our control group stimulus was previously used in prior research and did not produce changes in electrodermal activity and emotional stress (Molins et al. 2021), our sample reported a significant reduction in perceived stress levels with respect to the pretest. This should be considered as a limitation, but it is importance to notice that their change in bet acceptance had the opposite directionality to that observed in the experimental group. Furthermore, it is possible that some cognitive biases influenced the control group. Despite the limitation, the results observed are consistent with our purposes. Finally, we must mention some methodological issues. Due to COVID-19 restrictions and the high cumulative incidence, we had to opt for an online methodology to carry out our experiment. As described in Methods, we provided a step-by-step instructions to our participants trying to standardise as much as possible the procedure. We encouraged our sample to control their environment (e.g. controlling the lighting and reducing the presence of potential stressors) and choose a moment of the day when they felt most relaxed. However, we cannot not assure all procedural instructions were followed as we wanted. Thus, it would have been preferable to obtain complementary psychophysiological measures such as heart rate or electrodermal activity to strengthen our data, but it was not possible and would probably have been a source of stress given the sanitary situation. Further research should solve this.

Even so, our study provides substantial evidence of how emotional stress could affect decision-making under risk and puts the focus on how different sources of stressors may trigger different risky decisions. These results could be also relevant in the field of public health and clinical intervention. COVID-19 has become a drastic source of stress in general population over a long period of time. For its part, interoception seems to be an important modulating variable. The practical applications derived from this line of research can help general population as well as patients with impaired interoceptive processing—i.e. autism spectrum disorders—to make better decisions under emotional stress and uncertainty conditions.

## Supplementary Information

Below is the link to the electronic supplementary material.

Supplementary file1 (MP4 23512 kb)

Supplementary file2 (MP4 310153 kb)

Supplementary file3 (PDF 111 kb)

Supplementary file4 (PDF 117 kb)

Supplementary file5 (PDF 118 kb)

## Data Availability

The datasets generated during and/or analysed during the current study are available from the corresponding author on reasonable request.
