# Supplementary material for: COVID-19 stressor reduces risk taking: the role of trait interoception
Source: Cogn Process. 2023 Mar 28;24(3):353–60. doi: 10.1007/s10339-023-01134-4 (PMC10044126; doi:10.1007/s10339-023-01134-4)
Supplement: Supplementary file 3 — Supplementary file3 (PDF 111 kb) [file 10339_2023_1134_MOESM3_ESM.pdf]

Instructions for conducting the experiment: first phase

Welcome and thank you much for participating in this study. In this document you will find the access links and the step-by-step instructions to carry out the experiment. It is very important that you read the document carefully and do not alter the order of completion.

VERY IMPORTANT: at the beginning of each questionnaire you will be asked to enter a code. Remember that it must always be the same to be able to link the answers. We recommend that you use ONLY the first initial of your name and the LAST 3 digits of your ID card. For example: If my name is María José and my ID is 04160314J, the code would be "M314". You will be asked the same code to carry out the second phase of the study, so we recommend writing it down and saving it to avoid forgetting it.

BEFORE YOU BEGIN: it is important that you fill out the questionnaires at a moment of the day without distractions. Pick a place where you feel comfortable and don't have external pressures. Preferably use a computer, although it is not essential.

Considering the previous aspects, we can proceed to start this first part of the experiment.

REALIZATION INSTRUCTIONS

1. First, you must access the following questionnaire: https://forms.gle/Mx6ziL4BEVEQXWZy5 (you can access by clicking the link or copying it into your browser). Once there, you will be asked to enter the code (remember, **ALWAYS THE SAME**) and will be able to answer the questions.
2. Once the questionnaire is completed, in the farewell message, you will be offered a link to access the second and last questionnaire of this phase. You will be able to enter directly from it, but if there is a problem we will provide it here: https://forms.gle/yNrHBDLRVNoRTQp98 . You must enter **the access code** again and then, the test will begin.
3. After completing the second questionnaire, the first phase is over. You should not have to do nothing else, just wait for the second phase of the experiment to be available in the next few days.

We hope no problems during the process. If there is, feel free to contact us to let us know. Thank you very much for participating and see you in the second phase!
