# Supplementary material for: COVID-19 stressor reduces risk taking: the role of trait interoception
Source: Cogn Process. 2023 Mar 28;24(3):353–60. doi: 10.1007/s10339-023-01134-4 (PMC10044126; doi:10.1007/s10339-023-01134-4)
Supplement: Supplementary file 5 — Supplementary file5 (PDF 118 kb) [file 10339_2023_1134_MOESM5_ESM.pdf]

Instructions for conducting the experiment: second phase

Welcome and thank you for participating in this study. In this document you will find the access links and step-by-step instructions to perform the experiment. It is very important that you read the document carefully and do not alter the order of completion.

VERY IMPORTANT: at the beginning of each questionnaire, you will be asked to enter a code. Remember that it **MUST BE THE SAME one you used during the first phase** to be able to link your answers.

BEFORE YOU BEGIN: It is important that you complete the test at a moment of day when there are no distractions and you have the necessary time (**THIS PHASE REQUIRES MORE TIME** than the previous). Choose a place where you feel comfortable and do not have external pressures. In this case, you should preferably use a **computer** and it is highly recommended to have **headphones** available. Use an appropriate level of lighting: try not to be in the dark or that having an excess of light that can disturb you.

Taking into account the previous aspects, we can give way to this first part of the experiment.

REALIZATION INSTRUCTIONS

1. First, you will have to fill out a questionnaire. As you already know, **you will be asked for the code** and after adding it, you will be able to complete it. You can access the questionnaire from here: https://forms.gle/mhhLKZkmpi3qGcKx7
2. After completing this first questionnaire, you should watch a video file. It is important to watch it through your computer. If you have headphones, connect them at medium-high volume without being annoying (configure it at the volume in which you would listen to music). Stand at an approximate distance of about 40-50cm from the screen and access the link to see the video. You should watch it without interruptions (**FULL SCREEN**). Once finished, start the following steps of the experiment without interruptions. The link to access the video file is the following: https://drive.google.com/file/d/1xBXg_gzs4AHQVcWdLNBK8WD_gfMHJjfD/view?usp=sharing
3. Once you have seen the video, and without interruptions, you will have to complete the last two questionnaires. To avoid wasting time and distractions **, we recommend you to open them before in order to be ready**. Perform them **in the order** in which they are they presented to you.
   Questionnaire 1: https://forms.gle/zYPvwmGG49NKggKz7
   Questionnaire 2: https://forms.gle/6gdv58kksMZq59fy5

Once the questionnaires are completed, you will have finished this second phase and the experiment will have concluded. We really appreciate your participation and effort dedicating time to it. Thank you very much and happy week!
